# Glioma stem cell-derived exosomal miR-944 reduces glioma growth and angiogenesis by inhibiting AKT/ERK signaling

**DOI:** 10.18632/aging.203243

**Published:** 2021-07-07

**Authors:** Jianxin Jiang, Jun Lu, Xiaolin Wang, Bing Sun, Xiaoxing Liu, Yasuo Ding, Guangzhong Gao

**Affiliations:** 1 Department of Neurosurgery, Taizhou People’s Hospital, Taizhou 225300, Jiangsu, P.R. China

**Keywords:** glioma stem cells, angiogenesis, exosome, microRNA-944, VEGFC

## Abstract

In this study, we investigated the regulatory role of exosomal microRNA-944 (miR-944) derived from glioma stem cells (GSCs) in glioma progression and angiogenesis. Bioinformatics analysis showed that miR-944 levels were significantly lower in high-grade gliomas (HGGs) than low-grade gliomas in the Chinese Glioma Genome Atlas and The Cancer Genome Atlas datasets. The overall survival rates were significantly shorter for glioma patients expressing low miR-944 levels than high miR-944 levels. GSC-derived exosomal miR-944 significantly decreased *in vitro* proliferation, migration, and tube formation by human umbilical vein endothelial cells (HUVECs). Targetscan and dual luciferase reporter assays demonstrated that miR-944 directly targets the 3’UTR of VEGFC. *In vivo* mouse studies demonstrated that injection of agomiR-944 directly into tumors 3 weeks after xenografting glioma cells significantly reduced tumor growth and angiogenesis. GSC-derived exosomal miR-944 significantly reduced VEGFC levels and suppressed activation of AKT/ERK signaling pathways in HUVECs and xenograft glioma cell tumors. These findings demonstrate that GSC-derived exosomal miR-944 inhibits glioma growth, progression, and angiogenesis by suppressing VEGFC expression and inhibiting the AKT/ERK signaling pathway.

## INTRODUCTION

Glioma is the most common cancer of the central nervous system, and accounts for nearly 80 percent of all primary brain tumors [1, 2]. Glioma is a highly aggressive and invasive cancer characterized by poor prognosis and high mortality rates [3, 4]. According to the World Health Organization (WHO) criteria, glioma is classified into four grades: (1) pilocytic astrocytoma or grade I; (2) diffuse astrocytoma or grade II; (3) anaplastic astrocytoma or grade III; and (4) glioblastoma (GBM) or grade IV [5]. Surgery, chemotherapy, and radiotherapy are the main treatment strategies currently available for glioma patients [6, 7]. Despite advances in treatment strategies, prognosis and survival rates of patients with glioma remain unsatisfactory [8]. Cancer stem cells (CSCs) are highly resistant to chemotherapy and radiotherapy [9]. Glioma stem cells (GSCs) are associated with resistance to chemo- and radio-therapies and high recurrence rates of gliomas [10, 11]. GSCs promote glioma growth, progression, and angiogenesis [12]. Angiogenesis plays a crucial role in the development of gliomas because the tumor vasculature is necessary for supplying nutrients and oxygen to sustain glioma cell growth and survival [13]. However, the role of GSCs in angiogenesis is not fully understood.

Exosomes are small extracellular vesicles with sizes between 50–150 nm that are secreted by most cell types including stem cells and cancer cells [14, 15]. Exosomes serve as vehicles for transporting growth factors, signaling lipids, mRNAs, micro-RNAs (miRNAs), and long noncoding RNAs (lncRNAs) to recipient cells [16]. MiRNAs are small non-coding RNAs (20-24 nucleotides in length) that repress gene expression by binding to the 3′ UTR of their target mRNAs [17]. MiRNAs play a critical role in growth and progression of gliomas [18]. Exosomal miRNAs regulate proliferation, apoptosis, invasion, and angiogenesis of several types of tumor cells [19]. Wang et al. reported that exosomal miR-26a derived from the GSCs promotes angiogenesis of human brain microvascular endothelial cells in glioma patients [20]. Exosomal miRNAs also serve as potential diagnostic and prognostic biomarkers of gliomas [21]. Therefore, identifying differentially expressed miRNAs (DEMs) is critical in further understanding the pathogenesis and prognosis of gliomas.

In the present study, we compared miRNA expression profiles of low-grade glioma (LGG) and HGG patients from the Chinese Glioma Genome Atlas (CGGA) and The Cancer Genome Atlas (TCGA) databases to identify DEMs. Our data demonstrated that low expression of miR-944 was closely associated with poor survival and microvascular density in glioma patients. Therefore, we investigated potential underlying mechanisms by which exosomal miR-944 derived from GSCs regulated glioma angiogenesis and progression using HUVECs *in vitro* and *in vivo* xenograft glioma model mice.

## RESULTS

### MiR-944 is downregulated in glioma cells and associated with angiogenesis

**Figure 1:**
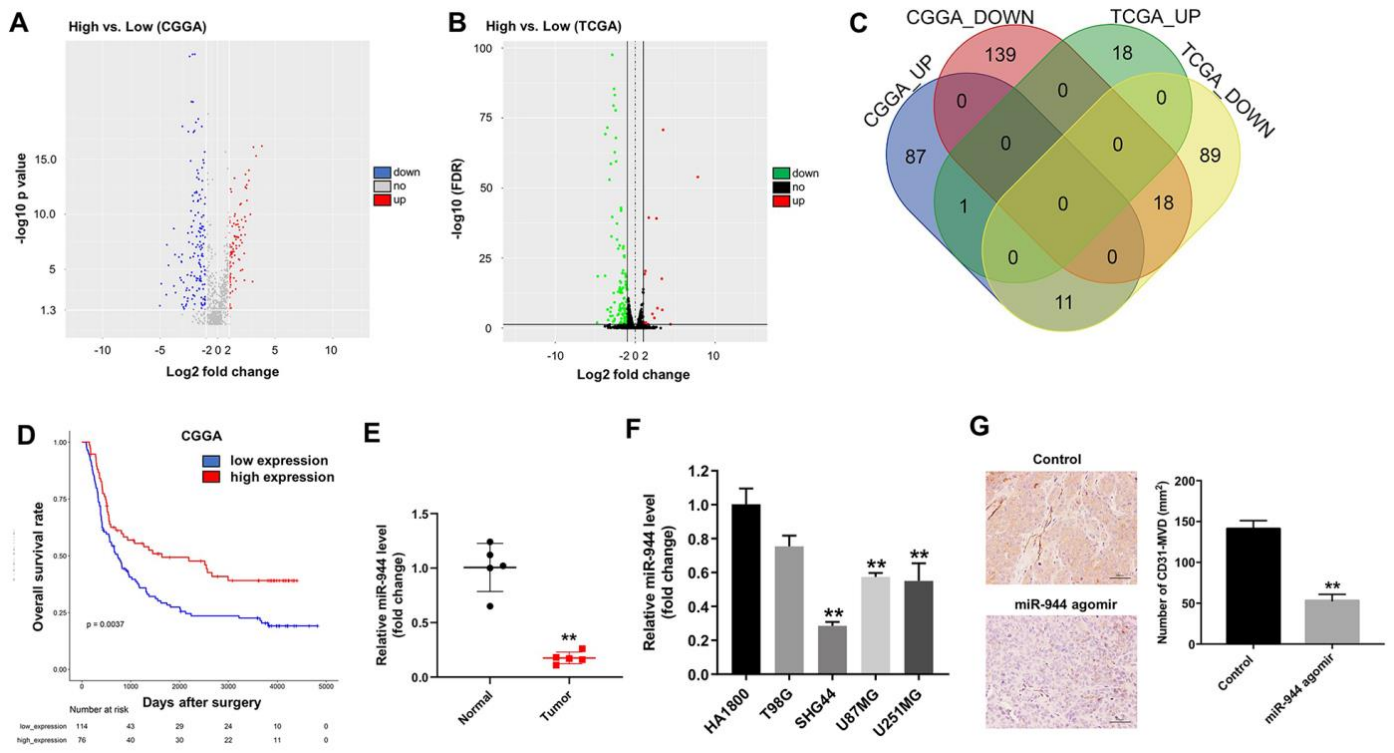
**Identification of differentially expressed miRNAs in gliomas from CGGA and TCGA datasets.** (**A**, **B**) Volcano plots show upregulated (red dots) and downregulated (blue dots) miRNAs in the glioma datasets from (**A**) CGGA and (**B**) TCGA databases. (**C**) Venn diagram shows the numbers of upregulated and downregulated miRNAs or DEMs that are common to both CGGA and TCGA datasets. (**D**) Kaplan-Meier survival curves show overall survival of glioma patients with low (n=114; blue line) or high (n=76; red line) miR-944 expression in the CGGA dataset. (**E**) RT-qPCR analysis shows miR-944 expression in glioma tissues and adjacent normal tissues. **P < 0.01 vs. Normal group. (**F**) RT-qPCR analysis shows miR-944 expression in HA1800 cells and human glioma cell lines, T98G, SHG44, U87MG, and U251MG. **P < 0.01 compared to the HA1800 group. (**G**) Immunohistochemical (IHC) staining results show CD31 staining of xenograft glioma cell tumor tissues derived from subcutaneous injections of SHG44 cells into nude mice. As shown, positive CD31 staining shows microvessel density in control, agomiR-944- or agomiR-NC-injected xenograft glioma cell tumors at 3 weeks. The tumors were directly injected with 50 nM agomiR-944 or agomiR-NC, twice every week for 3 weeks. **P < 0.01 vs. control group.

We analyzed the miRNA sequencing data for low-grade glioma (LGG) and high-grade glioma (HGG) patients from CGGA and TCGA databases. The results showed 256 DEMs (99 up-regulated and 157 down-regulated) in the CGGA-glioma dataset (Figure 1A) and 137 DEMs (19 up-regulated and 118 down-regulated) in the TCGA-glioma dataset (Figure 1B). As shown in the Venn diagram in Figure 1C, 1 up-regulated miRNA and 18 down-regulated miRNAs were common among the CGGA and TCGA datasets. Kaplan-Meier survival curve analysis showed that overall survival rates of glioma patients in the CGGA dataset with low miR-944 levels (n=114) were significantly shorter than those with higher miR-944 levels (n=76) (Figure 1D). In addition, miR-944 level was significantly upregulated in glioma tissues compared with that in adjacent normal tissues (Figure 1E). Next, we examined the levels of miR-944 in four human glioma cell lines, T98G, SHG44, U87MG, and U251MG in comparison with the human cortical astrocyte cell line, HA1800. RT-qPCR results showed that miR-944 levels were significantly downregulated in SHG44, U87MG, and U251MG cells compared to the HA1800 cells (Figure 1E). Since the expression of miR-944 was lowest in SHG44 cells, we used them for subsequent experiments. Moreover, IHC results demonstrated that microvessel density (MVD) was significantly reduced in the xenograft glioma tumors derived from the miR-944 agomir group mice compared to the agomiR-NC group mice (Figure 1F). These data suggested that miR-944 expression regulated angiogenesis in gliomas.

### Overexpression of miR-944 inhibited proliferation, migration and angiogenesis of HUVECs

**Figure 2:**
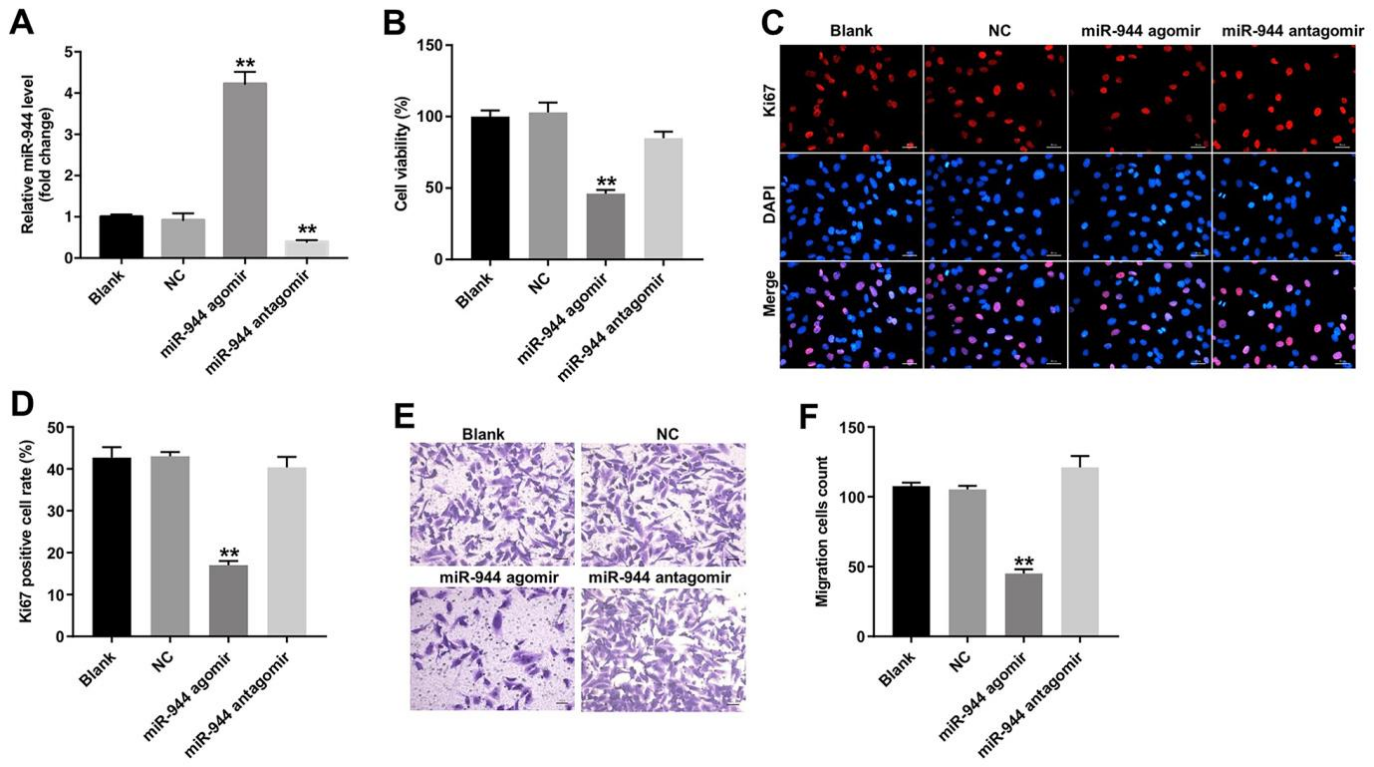
**Overexpression of miR-944 inhibits proliferation and migration of HUVECs.** (**A**) RT-qPCR analysis shows miR-944 expression in blank control, and agomiR-NC, agomiR-944, and antagomiR-944-transfected HUVECs. (**B**) CCK-8 assay results show viability of blank control, agomiR-NC-, agomiR-944-, and antagomiR-944-transfected HUVECs. (**C**, **D**) Immunofluorescence assay results demonstrate staining of blank control, and agomiR-NC-, agomiR-944-, and antagomiR-944-transfected HUVECs with anti-Ki67 antibody to determine the percentage of Ki67-positive HUVECs. Note: Ki67 is a proliferation marker. (**E**, **F**) Transwell migration assay results demonstrate the migration ability of blank control, and agomiR-NC-, agomiR-944-, and antagomiR-944-transfected HUVECs. **P < 0.01 vs. NC group. HUVECs, human umbilical vein endothelial cells; NC, negative control.

**Figure 3:**
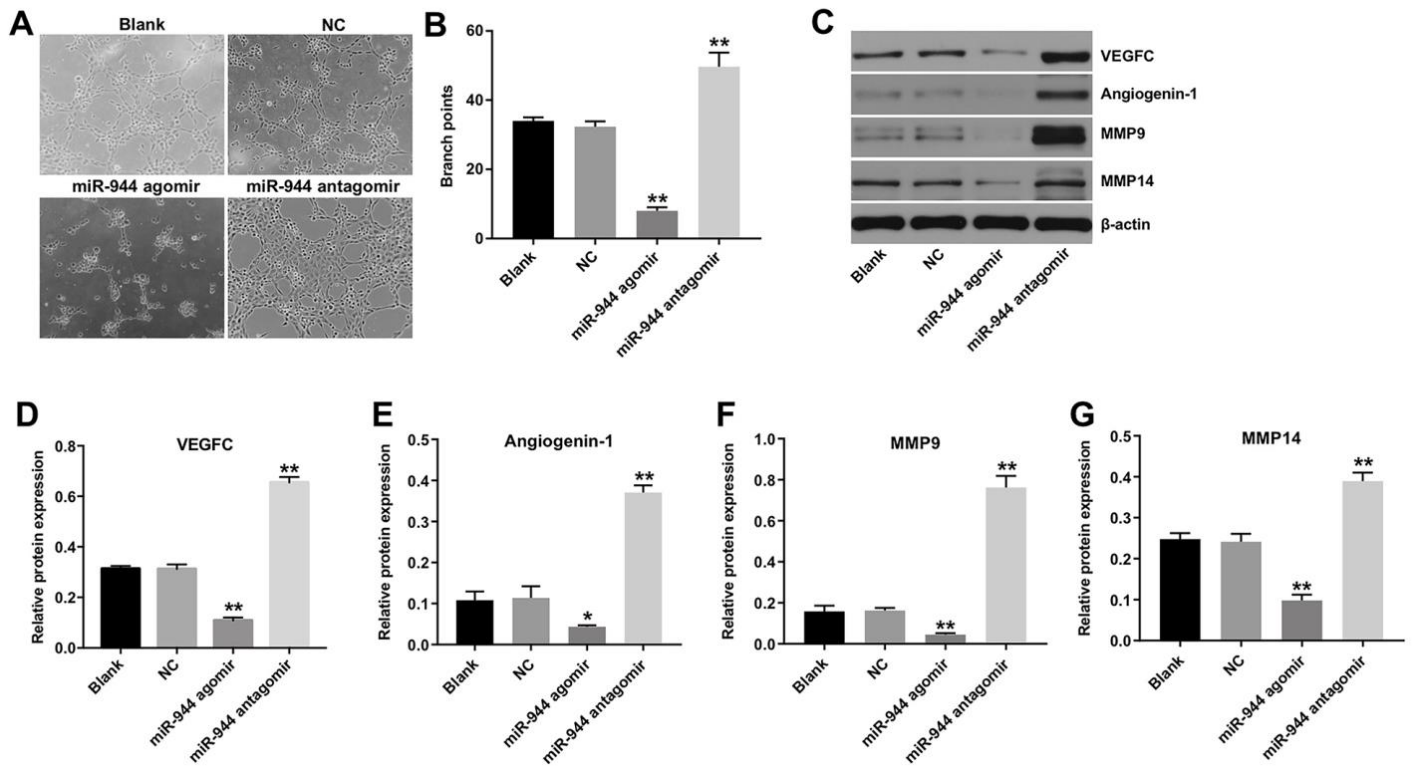
**Overexpression of miR-944 inhibits angiogenesis of HUVECs.** (**A**, **B**) Tube formation assay results show the number of branch points as an index of angiogenesis in blank control, and agomiR-NC-, agomiR-944-, and antagomiR-944-transfected HUVECs. (**C**–**G**) Western blot analysis shows the relative expression levels of (**C**, **D**) VEGF, (**C**, **E**) angiogenin-1, (**C**, **F**) MMP9, and (**C**, **G**) MMP14 proteins in blank control, and agomiR-NC, agomiR-944, and antagomiR-944-transfected HUVECs. *P < 0.05, **P < 0.01 vs. NC group. NC, negative control; HUVECs, human umbilical vein endothelial cells.

We then investigated the effects of miR-944 overexpression or silencing on the angiogenesis of HUVECs. HUVECs transfected with miR-944 agomir showed significantly higher levels of miR-944 compared to agomir-NC-transfected HUVECs; moreover, HUVECs transfected while miR-944 antagomir showed significantly lower levels of miR-944 (Figure 2A). Furthermore, miR-944 overexpression significantly reduced viability and proliferation (Figure 2B–2D) and *in vitro* migration (Figure 2E, 2F) of HUVECs compared to the agomir-NC-transfected HUVECs. Moreover, *in vitro* tube formation assay results demonstrated that miR-944 overexpression significantly reduced branch points and miR-944 silencing significantly increased branch point formation in HUVECs (Figure 3A, 3B). We also observed that overexpression of miR-944 significantly decreased VEGF, angiogenin-1, MMP9, and MMP14 protein levels, whereas, miR-944 knockdown significantly increased VEGF, angiogenin-1, MMP9, and MMP14 protein levels in HUVECs (Figure 3C–3G). These results demonstrated that overexpression of miR-944 suppressed *in vitro* proliferation, migration and angiogenesis of HUVECs.

### MiR-944 is downregulated in GSCs

**Figure 4:**
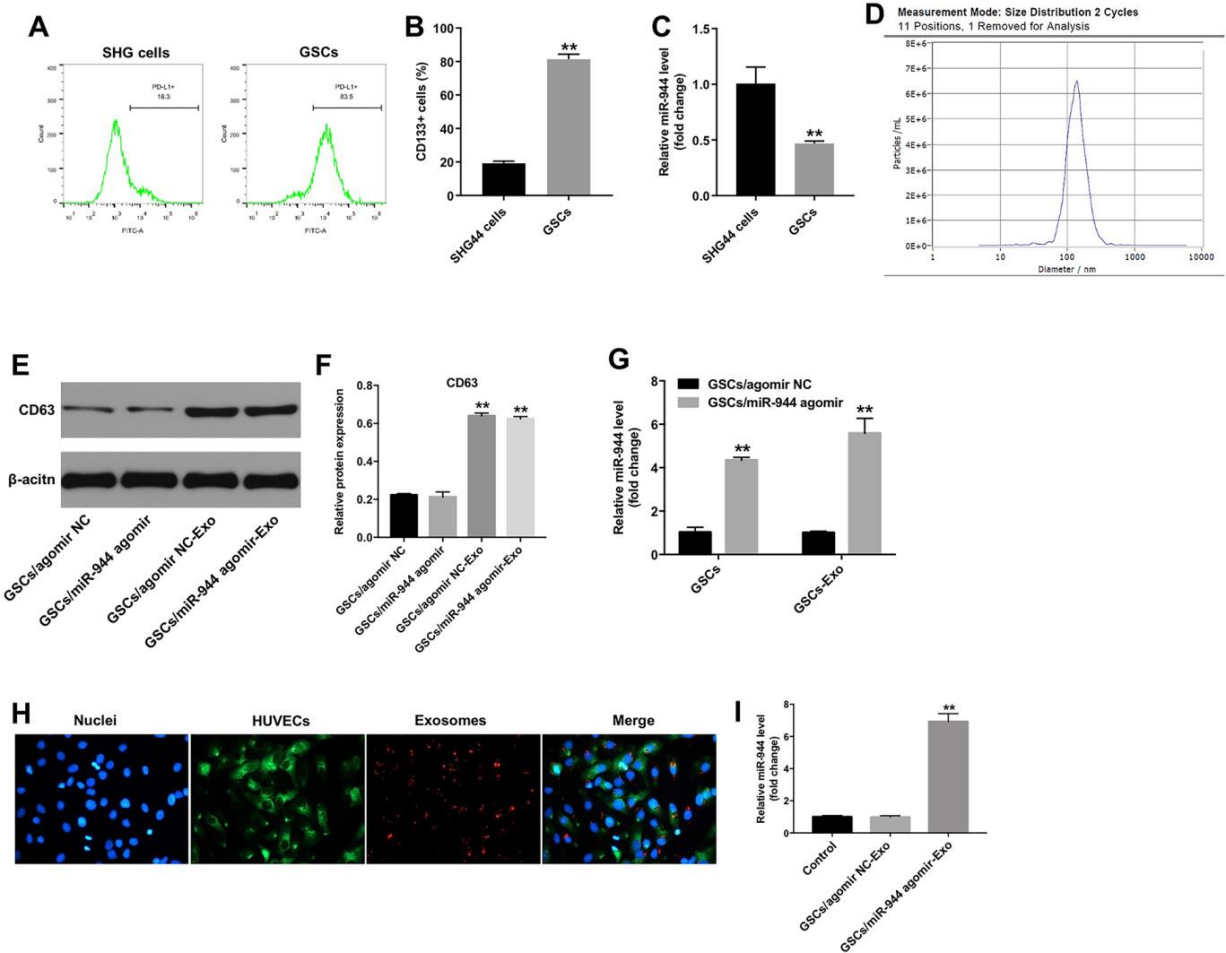
**Exosomes transport GSC-derived miR-944 to HUVECs.** (**A**, **B**) Flow cytometry analysis shows isolation and enrichment of CD133^+^ GSCs from SHG44 cells. ** denotes P < 0.01 vs. SHG44 cell group. (**C**) RT-qPCR analysis shows miR-944 levels in SHG44 cells and CD133^+^ GSCs. **P < 0.01 vs. SHG44 cell group. (**D**) NTA results show mean diameter of exosomes isolated from the conditioned medium of agomiR-944-transfected GSCs. (**E**, **F**) Western blotting analysis shows CD63 levels in agomir-NC-transfected GSCs (GSC/agomiR-NC), agomiR-944-transfected GSCs (GSC/agomiR-944), GSC/agomir-NC-derived exosomes (GSC/agomiR-NC-Exo), and GSC/agomiR-944 agomir-derived exosomes (GSC/agomiR-944-Exo). **P < 0.01 vs. the GSC/agomiR-NC group. (**G**) RT-qPCR analysis shows miR-944 expression levels in GSC/agomiR-NC, GSC/agomiR-944, GSC/agomiR-NC-Exo, and GSC/agomiR-944-Exo. **P < 0.01 vs. the GSC/agomir-NC group. (**H**) Confocal microscopy images show exosomes and their uptake into HUVECs. Exosomes are stained with PKH67 (red color); HUVECs are stained with phalloidin-FITC (green color); nuclei are stained with DAPI (blue color). (**I**) RT-qPCR analysis shows miR-944 levels in HUVECs co-cultured with GSC/agomiR-NC-Exo or GSC/agomiR-944-Exo. **P < 0.01 vs. the GSC/agomir-NC-exo group. GSCs, glioma stem cells; HUVECs, human umbilical vein endothelial cells.

GSCs express CD133 and display stem cell properties such as self-renewal and multi-lineage differentiation [22, 23]. Therefore, we isolated CD133^+^ GSCs from SHG44 cells using flow cytometry. The percentage of CD133^+^ cells was 81.4 % in the GSCs and 19.2% in the SHG-44 cells (Figure 4A, 4B). RT-qPCR analysis showed that miR-944 levels were significantly reduced in the GSCs compared to the SHG44 cells (Figure 4C).

### GSCs transfer miR-944 to HUVECs by exosomes

Several studies have shown that tumor cell-derived exosomal miRNAs regulate functions of recipient cells [24]. Therefore, we investigated if GSC-derived miR-944 was transferred to the HUVECs via exosomes. Towards this, we overexpressed miR-944 in the GSCs (GSC/agomiR-944) and isolated exosomes from the conditioned medium (CM) of GSC/agomiR-944. We performed NTA analysis to determine to characterize the isolated exosomes (Figure 4D). Western blot analysis showed enrichment of CD63 in the purified exosomes (Figure 4E, 4F). RT-qPCR analysis showed that miR-944 levels were significantly higher in agomiR-944-transfected GSCs and GSC/agomiR-944-derived exosomes compared to agomiR-NC-transfected GSCs (GSC/agomiR-NC) and GSC/agomir-NC-derived exosomes, respectively (Figure 4G).

Next, we incubated HUVECs with PKH67-labeled GSC-derived exosomes (GSC-exosomes) for 24 h and observed the PKH67 lipid dye in the recipient HUVEC cells (Figure 4H). This demonstrated that GSC-exosomes were internalized by the HUVECs. We further observed that miR-944 expression levels were significantly higher in HUVECs incubated with GSC/miR-944-agomir-derived exosomes compared to HUVECs incubated with GSC/agomir-NC-derived exosomes (Figure 4I). These data demonstrated that exosomes delivered GSC-derived miR-944 to the HUVECs.

### GSCs-derived exosomal miR-944 suppressed proliferation, migration, angiogenesis of HUVECs

**Figure 5:**
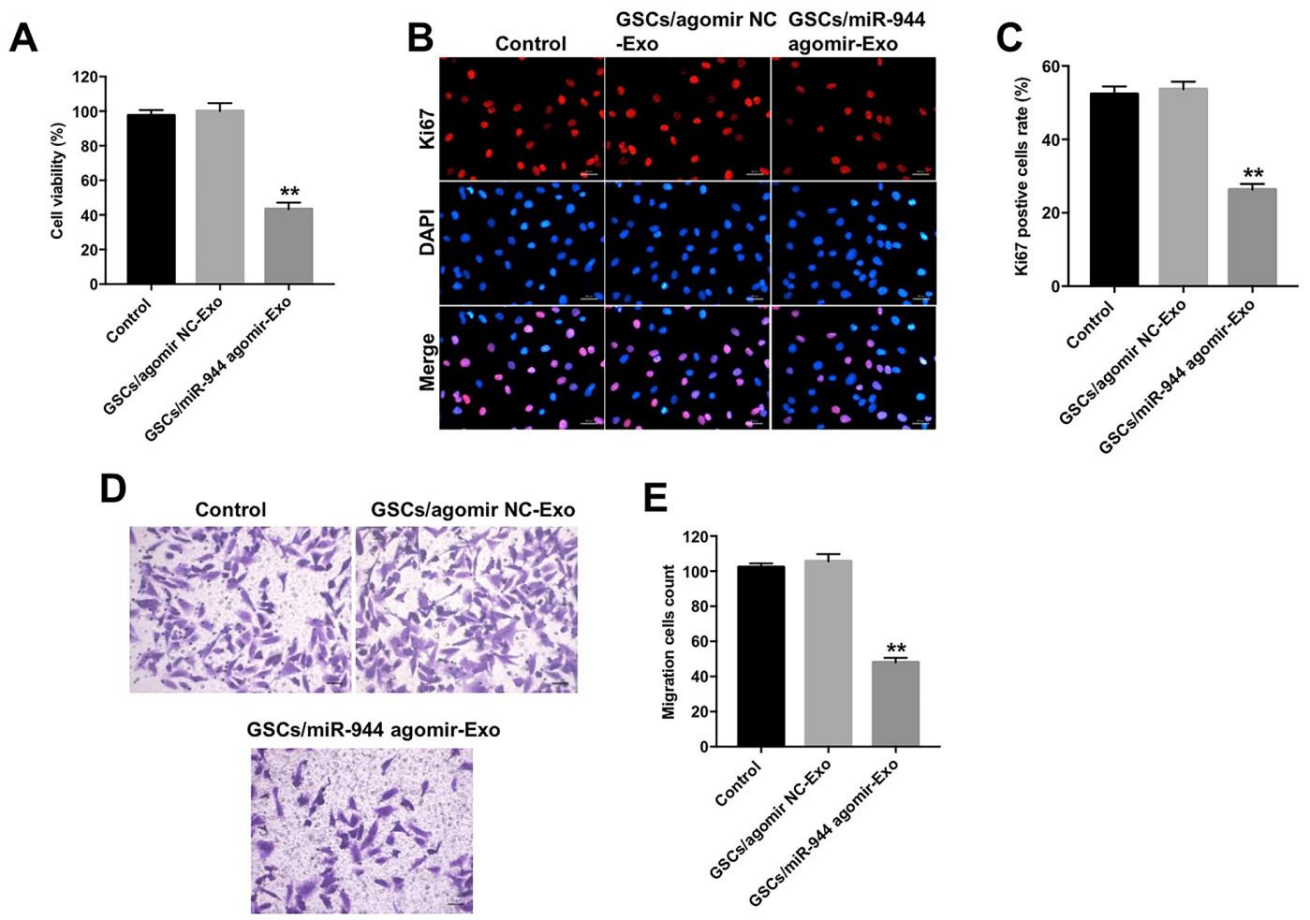
**Exosomal miR-944 derived from GSCs suppresses proliferation and migration of HUVECs.** (**A**) CCK-8 assay results show viability of HUVECs co-cultured with GSCs/agomiR-NC-Exo and GSCs/agomiR-944-Exo for 24 h. (**B**, **C**) Immunofluorescence assay results show the proliferation status of HUVECs co-cultured with GSCs/ agomiR-NC-Exo and GSCs/agomiR-944-Exo for 24 h based on Ki-67 staining. (**D**, **E**) Transwell assay results show the migration ability of HUVECs co-cultured with GSCs/agomiR-NC-Exo and GSCs/agomiR-944-Exo for 24 h. **P < 0.01 vs. the GSCs/agomiR-NC-exo group. GSCs, glioma stem cells; HUVECs, human umbilical vein endothelial cells.

**Figure 6:**
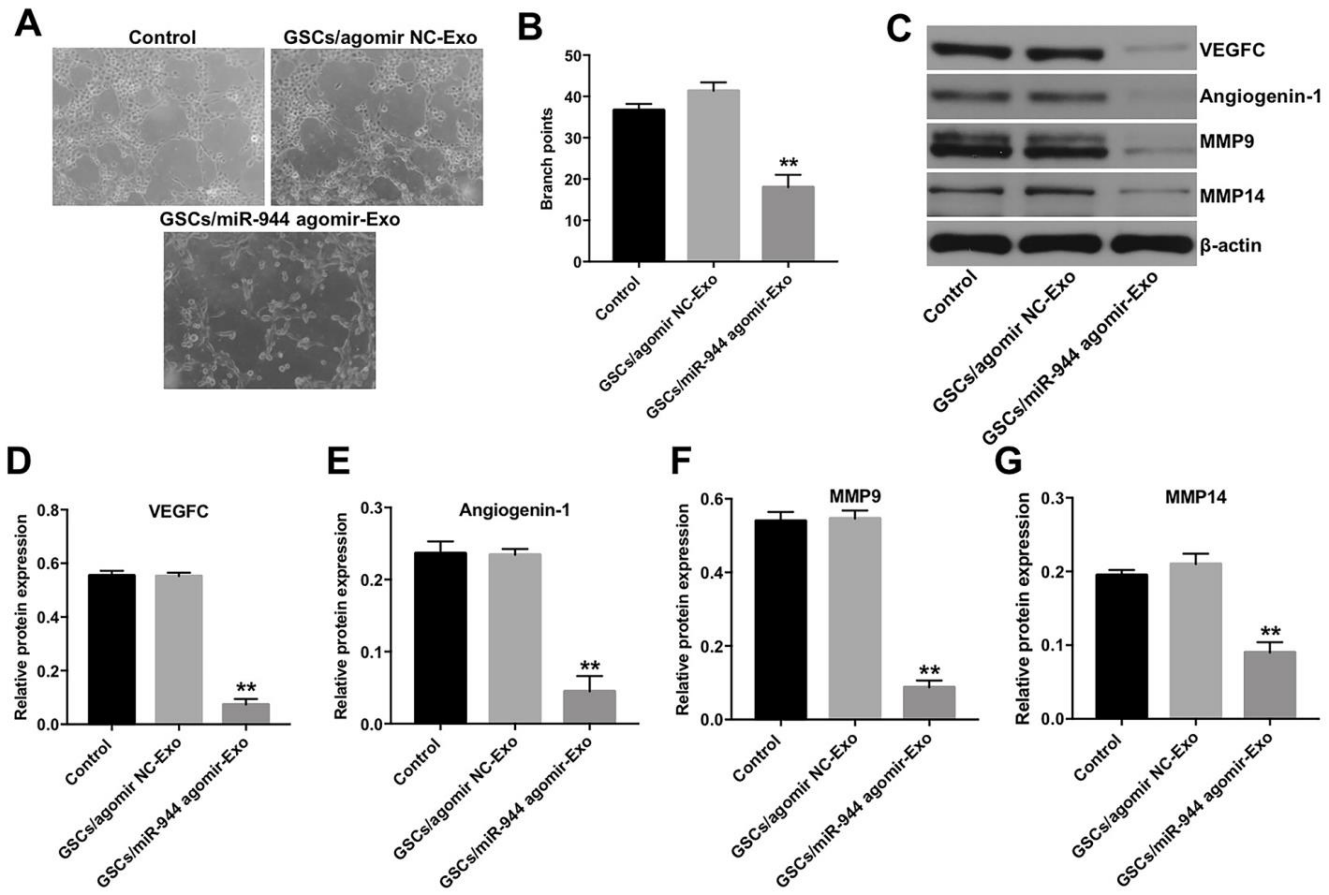
**Exosomal miR-944 derived from GSCs suppresses angiogenesis of HUVECs.** (**A**, **B**) Tube formation assay results show the number of branch points as an index of angiogenesis in HUVECs co-cultured with GSC/agomiR-NC-Exo and GSC/agomiR-944-Exo for 24 h. (**C**–**G**) Western blot analysis shows levels of (**C**, **D**) VEGF, (**C**, **E**) angiogenin-1, (**C**, **F**) MMP9, and (**C**, **G**) MMP14 in HUVECs co-cultured with GSC/agomiR-NC-Exo and GSC/agomiR-944-Exo for 24 h. β-actin was used as an internal control. **P < 0.01 vs. the GSC/agomiR-NC-Exo group. GSCs, glioma stem cells; HUVECs, human umbilical vein endothelial cells.

Next, we analyzed the biological role of exosomal miR-944 by co-culturing HUVECs with exosomes derived from GSC/agomiR-944 or GSC/agomiR-NC. We observed significantly reduced viability, proliferation, and migration of HUVECs co-cultured with GSC/agomiR-944 exosomes compared to those co-cultured with GSC/agomiR-NC exosomes (Figure 5A–5E). Moreover, tube formation and branching were significantly reduced in HUVECs co-cultured with GSC/miR-944 exosomes (Figure 6A, 6B). Western blot analysis showed that VEGF, angiogenin-1, MMP9, and MMP14 protein levels were significantly reduced in HUVECs co-cultured with GSC/miR-944 exosomes compared to those co-cultured with GSC/agomiR-NC exosomes (Figure 6C–6G). These data demonstrated that exosomal miR-944 derived from GSCs significantly reduced viability, proliferation, migration, and angiogenesis of HUVECs.

### GSC-derived exosomal miR-944 suppressed proliferation and angiogenesis of HUVECs via VEGFC

**Figure 7:**
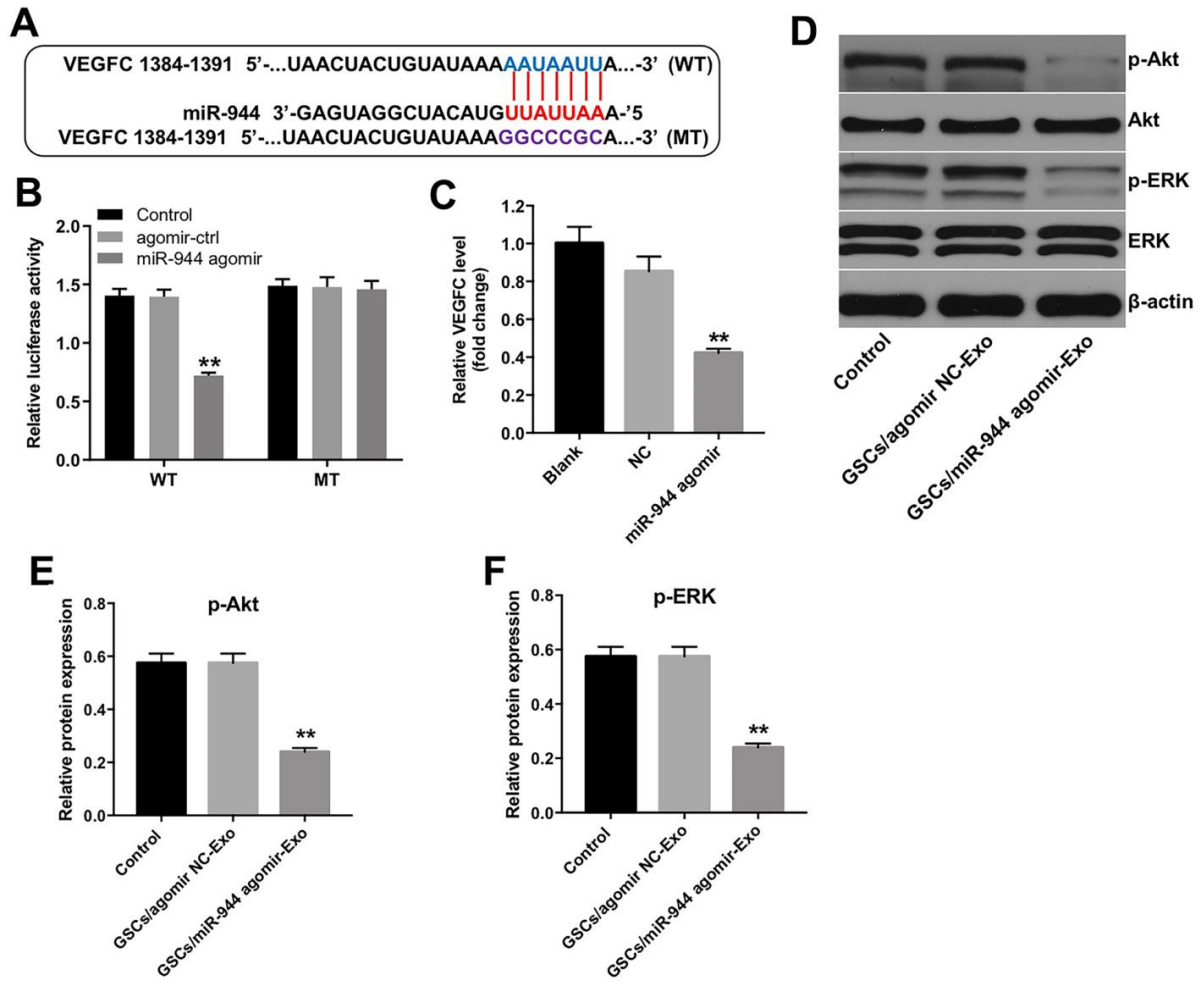
**Exosomal miR-944 derived from GSCs suppresses angiogenesis of HUVECs by targeting VEGFC.** (**A**) Targetscan analysis results show miR-944 binding sites in the 3’UTR of VEGFC. The mutated miR-944 binding site in the 3’UTR of VEGFC is also shown. (**B**) Dual luciferase reporter assay results show relative luciferase activity in HUVECs co-transfected with agomiR-NC or agomiR-944 plus luciferase vector with VEGFC-3’UTR-WT or VEGFC-3’UTR-MUT. A blank control was also included. **P < 0.01 vs. the agomir-ctrl group. (**C**) RT-qPCR analysis shows VEGFC levels in HUVECs transfected with agomiR-NC or agomiR-944. **P < 0.01 vs. the agomiR-NC group. (**D**–**F**) Western blot analysis shows expression levels of p-Akt, Akt, p-ERK, and ERK in HUVECs co-cultured with GSC/agomiR-NC-Exo and GSC/agomiR-944-Exo for 24 h. The relative expression levels of p-Akt and p-ERK in HUVECs were normalized to total Akt and total ERK levels, respectively. **P < 0.01 vs. the GSC/agomiR-NC-Exo group. NC, negative control; GSCs, glioma stem cells; HUVECs, human umbilical vein endothelial cells.

Targetscan analysis showed that VEGFC was the potential target of miR-944 because the 3’UTR sequence of its mRNA contained a complementary miR-944 binding sequence (Figure 7A). Wang et al. reported that downregulation of VEGFC inhibited growth and angiogenesis of bladder cancer cells [25]. Dual luciferase reporter assay results showed that relative luciferase activity was significantly inhibited in HUVECs co-transfected with agomiR-944 and luciferase vector with VEGFC-WT-3’UTR, but was significantly high in HUVECs co-transfected with agomiR-944 and luciferase vector with VEGFC-MUT-3’UTR (Figure 7B). Moreover, VEGFC levels were significantly reduced in HUVECs transfected with agomiR-944 compared to those transfected with agomiR-NC (Figure 7C). These results confirmed that miR-944 suppressed VEGFC expression in HUVECs.

Next, CCK-8 and tube formation assays were performed to determine whether miR-944 exhibited antiangiogenic effects via downregulation of VEGFC. As shown in Supplementary Figure 1A, miR-944 agomir markedly inhibited the viability of HUVECs, whereas that effect was reversed by VEGFC overexpression. In addition, miR-944 agomir notably suppressed the tube formation of HUVECs, while that effect was reversed by VEGFC overexpression (Supplementary Figure 1B). These data verified that miR-944 inhibited the viability and angiogenesis of HUVECs via downregulation of VEGFC.

VEGFC promotes tumor growth and angiogenesis by activating Akt and ERK signaling pathways [26]. Therefore, we analyzed if exosomal miR-944 regulated Akt and ERK signaling pathways in HUVECs via VEGFC. Western blot analysis showed that the levels of phospho-Akt and phospho-ERK proteins were significantly reduced in HUVECs co-cultured with GSC/agomiR-944 exosomes compared to those co-cultured with GSC/agomiR-NC exosomes (Figure 7D–7F). These data demonstrated that GSC-derived exosomal miR-944 decreased proliferation and angiogenesis of HUVECs by suppressing VEGFC and inhibiting the activation of the Akt/ERK signaling pathways.

### Overexpression of miR-944 inhibits *in vivo* xenograft glioma growth and angiogenesis

**Figure 8:**
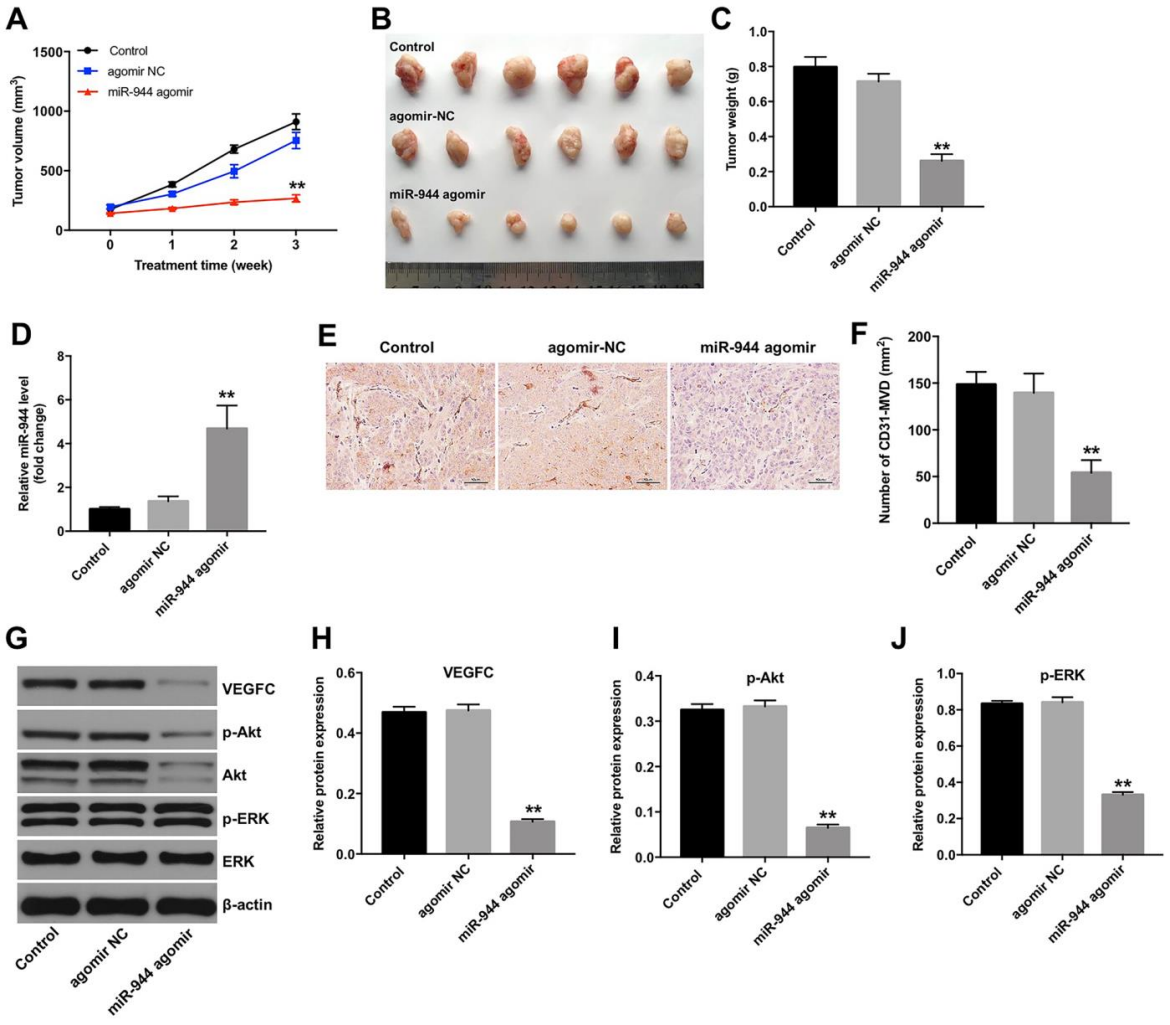
**Overexpression of miR-944 inhibits *in vivo* xenograft glioma cell tumor growth and angiogenesis.** (**A**) The line graph shows tumor volumes on weeks 1-3 in control, agomiR-NC, and agomiR-944 group nude mice. (**B**) The photographs show xenograft glioma cell tumors harvested at four weeks from control, agomiR-NC, and agomiR-944 group nude mice. (**C**) The histogram shows the weights of xenograft glioma cell tumors harvested at four weeks from control, agomiR-NC, and agomiR-944 group nude mice. (**D**) RT-qPCR analysis shows miR-944 levels in the xenograft glioma cell tumor tissues harvested from nude mice belonging to the control, agomiR-NC, and agomiR-944 groups. (**E**, **F**) IHC staining results show CD31-stained xenograft glioma cell tumor tissues harvested from nude mice belonging to the control, agomiR-NC, and agomiR-944 groups. Microvessel density (MVD) was analyzed based on CD31^+ve^ staining. (**G**–**J**) Western blot analysis shows the levels of VEGFC, p-Akt, Akt, p-ERK, and ERK proteins in the xenograft glioma cell tumor tissues harvested from control, agomiR-NC, and agomiR-944 groups. VEGFC, p-Akt, and p-ERK levels were normalized to β-actin, Akt and ERK levels, respectively. **P < 0.01 vs. the agomiR-NC group; NC, negative control; MVD, microvessel density; IHC, immunohistochemistry.

Next, we investigated the role of miR-944 in tumor growth and angiogenesis *in vivo*. Towards this, we subcutaneously injected GSCs into nude mice and then injected agomiR-NC or agomiR-944 into the xenograft glioma tumors. Tumor volume and weight was significantly reduced in the agomiR-944 group compared to the agomiR-NC group (Figure 8A–8C). RT-qPCR analysis showed significantly higher levels of miR-944 in the xenograft tumor tissues from the agomiR-944 group compared to those from the agomiR-NC group (Figure 8D). Moreover, IHC assay results with anti-CD31 antibodies showed significant reduction in CD31^+^ microvessel density (MVD) in the xenograft tumor tissues from the agomiR-944 group compared to those from the agomiR-NC group (Figure 8E, 8F). We also observed significantly reduced levels of VEGFC, p-Akt and p-ERK proteins in the xenograft tumor tissues from the agomiR-944 group compared to those from the agomiR-NC group (Figure 8G–8J). These results confirmed that miR-944 overexpression inhibited *in vivo* glioma growth and angiogenesis.

## DISCUSSION

The prognosis of malignant glioma remains poor despite advances in diagnostic techniques and treatment strategies [27]. Blood vessel abnormalities including poor perfusion and vascular leakage promote gliomagenesis and poor response to available chemotherapies [28, 29]. However, molecular mechanisms underlying angiogenesis in gliomas remain largely unknown. In this study, we demonstrated that miR-944 levels were significantly downregulated in glioma cells. Glioma patients with low levels of miR-944 were associated with poor prognosis. We also demonstrated that MVD was significantly reduced in xenograft glioma tumors expressing higher levels of miR-944. This suggested a negative correlation between miR-944 levels and glioma angiogenesis. We also demonstrated that downregulation of miR-944 increased angiogenesis of HUVECs, whereas, upregulation of miR-944 decreased angiogenesis of HUVECs. Previous studies have shown that the level of miR-944 was significantly upregulated in endometrial and breast cancers [30, 31]. And it was notably downregulated in lung and colorectal cancers [32, 33]. In this study, we found that miR-944 level was markedly decreased in glioma tissues. Taken together, miR-944 may function as an oncogene or a tumor suppressor, depending on the tumor types.

The concept of cancer stem cells (CSCs) was refined at the beginning of the 21st century, and CSCs play a central role in the initiation of cancer [34]. GSCs display properties of normal stem cells such as capacity for multipotent differentiation and indefinite self-renewal [29, 35]. Moreover, GSCs are localized in close proximity to blood vessels in the tumor microenvironment [36]. The vascular endothelial cells secrete extracellular factors that promote expansion of GSCs [37]. GSCs induce neovascularization by secreting factors that promote proliferation and tube formation of vascular endothelial cells [38]. Previous studies have demonstrated that GSCs release extracellular vesicles (including exosomes) that contain pro-angiogenic proteins, mRNAs, and miRNAs, which are taken up by the brain microvascular endothelial cells [20, 36, 39]. Exosomes function as intercellular communicators during progression of several cancer types [40]. Sun et al. reported that exosomal miR-21 derived from GSCs promoted angiogenesis of endothelial cells [41]. Moreover, exosomal miR-26 derived from GSCs promoted tube formation of microvessel endothelial cells [20]. Our data showed that miR-944 was downregulated in GSCs. Moreover, overexpression of miR-944 in the GSCs increased the levels of exosomal miR-944. We also demonstrated that exosomes delivered GSC-derived miR-944 to the HUVECs. Exosomes with high miR-944 levels significantly inhibited proliferation and tube formation of HUVECs. Consistent with our finding, exosomal miR-9 from nasopharyngeal carcinoma cells inhibited migration and tube formation of endothelial cells [42]. Furthermore, overexpression of miR-944 inhibited *in vivo* xenograft glioma growth and angiogenesis. These data demonstrated that exosome-mediated transfer of miR-944 from GSCs to HUVECs promoted glioma progression.

MiRNAs exert their biological functions by inhibiting target gene expression levels [43]. Bioinformatics and functional analysis demonstrated that VEGFC was a direct target gene of miR-944. VEGFC is a major driver of lymphangiogenesis, and plays an important role in growth and progression of gliomas [44, 45]. Michaelsen et al. demonstrated that VEGFC promoted glioma cell survival, growth, and progression [45]. In this study, our data demonstrated that miR-944 decreased VEGFC expression in glioma cells. Yan et al. demonstrated that miR-944 inhibited growth and invasiveness of osteosarcoma cells by targeting VEGF [46]. Chen et al. showed that overexpression of miR-107 inhibited glioma angiogenesis by downregulating VEGF [47]. In our study, *in vivo* experiments showed that agomiR-944 injections into xenograft glioma tissues for 3 weeks significantly decreased expression of VEGFC in the glioma tissues. Moreover, GSC-derived miR-944 downregulated expression levels of VEGFC in HUVECs. Exosomes derived from agomiR-944-transfected GSCs inhibited proliferation, migration, and tube formation of HUVECs. These data demonstrated that exosomal miR-944 inhibited angiogenesis of HUVECs by downregulating VEGFC.

**Figure 9:**
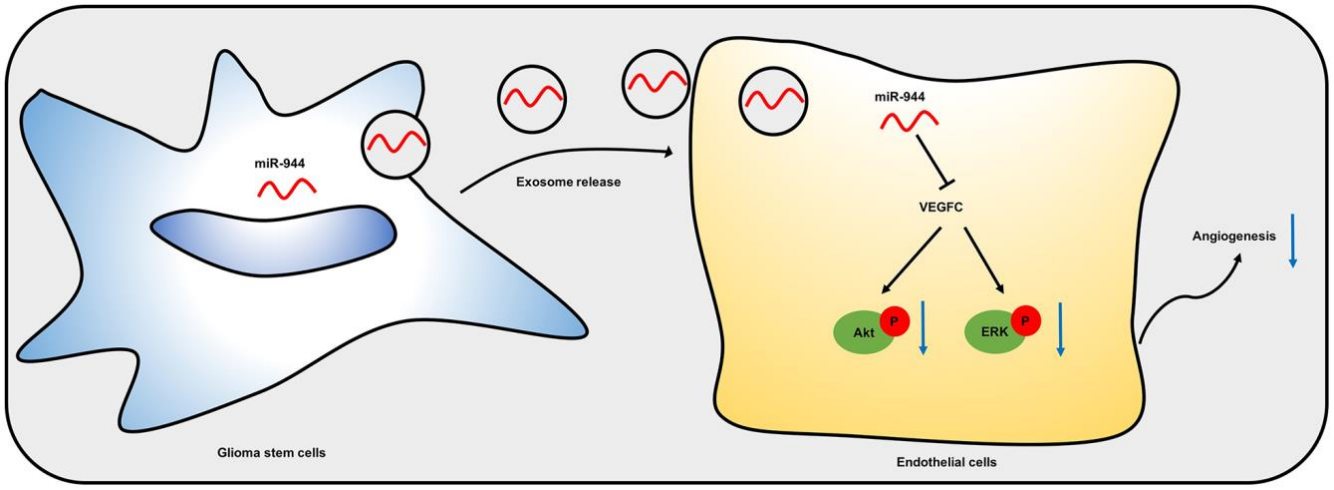
**A schematic model shows mechanistic role of miR-944 in glioma angiogenesis.** Exosomal miR-944 derived from GSCs reduces angiogenesis of HUVECs by inhibiting VEGFC expression and suppressing the activation of Akt/ERK signaling pathways.

Dai et al. reported that miR-24 overexpression promoted glioma angiogenesis by upregulating VEGF and activating the Akt signaling pathway [48]. Feng et al. showed that overexpression of miR-770 suppressed migration and invasion of glioma cells by inhibiting the PI3K/Akt signaling pathway [49]. Zheng et al. demonstrated that miR-15b and miR-152 decreased invasion and angiogenesis of glioma cells by inhibiting the MEK-ERK pathway [50]. We demonstrated that exosomal miR-944 inhibited activation of Akt and ERK in the HUVECs. Overall, our results suggested that exosomal miR-944 derived from the GSCs inhibited angiogenesis of HUVECs by suppressing VEGFC expression and significantly reducing the activation of Akt and ERK signaling pathways (Figure 9).

In conclusion, our study demonstrated that exosomes served as vehicles for transferring miR-944 from the GSCs to glioma cells. Moreover, GSC-derived exosomal miR-944 inhibited glioma angiogenesis by downregulating VEGFC and suppressing the activation of the AKT/ERK signaling pathways.

## MATERIALS AND METHODS

### Data collection and differential miRNA expression analysis

The miRNA expression data for the LGG and HGG tissues were downloaded from the CGGA and TCGA databases. R language was used to identify differentially expressed miRNAs (DEMs) using adjusted P value < 0.05 and |log2 (fold change)| > 2 as threshold parameters. The VennDiagram R package was used to identify common DEMs among the CGGA and TCGA datasets. Kaplan-Meier survival curve analysis was performed to determine prognostic significance of the DEMs between LGG and HGG tissues using the CGGA dataset. P<0.05 was considered statistically significant.

### Clinical samples

A total of five paired glioma tissue and adjacent normal tissues were obtained from the Taizhou People’s Hospital, between July 2019 and September 2020. None of these participants were subjected to adjuvant therapy before surgery. Written informed consent was obtained from all participants. This study was approved by the Institutional Ethical Committee of Taizhou People’s Hospital.

### Cell culture

We purchased human cortical astrocyte cell line, HA1800, human glioma cell lines (T98G, SHG44, U87MG, and U251MG), and human umbilical vein endothelial cells (HUVECs) from the Type Culture Collection of the Chinese Academy of Sciences (Shanghai, China). The cells were cultured in DMEM medium (Thermo Fisher Scientific) containing 10% FBS and 100 U/mL penicillin/streptomycin in a humidified incubator at 37° C and 5% CO_2_.

### Reverse transcription-quantitative polymerase chain reaction (RT-qPCR)

Total cellular RNA was extracted using TRIzol reagent (Thermo Fisher Scientific). Reverse transcription of total RNA samples was performed using EntiLink™ 1st Strand cDNA Synthesis Kit (ELK Biotechnology). Then, qPCR was performed using EnTurbo™ SYBR Green PCR SuperMix (ELK Biotechnology) in an ABI StepOne-Plus Real-Time Quantitative PCR System (Thermo Fisher Scientific). The sequences of primers used for qPCR are listed in Table 1. The data was analyzed using the 2^–ΔΔCT^ method. Actin and U6 were used as internal controls for VEGFC and miR-944, respectively.

**Table 1:** Primer sequences.

**Name**		**Primer sequences**
U6	Forward	5’-CTCGCTTCGGCAGCACAT-3’
	Reverse	5’-AACGCTTCACGAATTTGCGT-3’
miR-944	Forward	5’-AATTATTGTACATCGGATGAGCTC-3’
	Reverse	5’-CTCAACTGGTGTCGTGGAGTC-3’
Actin	Forward	5’-GTCCACCGCAAATGCTTCTA-3’
	Reverse	5’-TGCTGTCACCTTCACCGTTC-3’
VEGFC	Forward	5’-ACGAGCTACCTCAGCAAGACG-3’
	Reverse	5’-CTCCAGCATCCGAGGAAAAC-3’

### Cell transfections

We purchased agomiR-944, antagomiR-944, and non-specific control (agomiR-NC) expression vectors from Suzhou Ribo Life Science Co., Ltd (Jiangsu, China). The glioma cells were transfected with agomiR-NC, agomiR-944 or antagomiR-944 using Lipofectamine™2000 (Thermo Fisher Scientific) according to manufacturer’s recommendations. The transfected cells were cultured for 6 h in a humidified incubator maintained at 5% CO_2_ and 37° C. Then, the medium was replaced and cells were further cultured for another 48 h.

The pcDNA3.1-VEGF-C overexpression (VEGF-C-OE) plasmids were transfected into HUVECs by using Lipofectamine 2000 reagents. Later on, the transfected cells were selected with neomycin at 48 h.

### Cell Counting Kit-8 (CCK-8) assay

The cells (5000 cells/well) were cultured in a 96-well plate for specific time points. Then, 10 μL CCK-8 (Beyotime, Shanghai, China) was added to the wells. The cells were further incubated for 2 h at 37° C. The absorbance was measured at 450 nm using a microplate reader (Bio-Rad Laboratories, Inc.).

### Immunofluorescence

The cells were fixed in 4% paraformaldehyde for 15 min, and blocked with 3% BSA for 1 h. Then, the cells were incubated overnight at 4° C with anti-Ki67 antibody (1: 100; Abcam; Cambridge, MA, USA) followed by incubation with horseradish peroxidase-conjugated secondary antibody (Abcam) for 1 h at room temperature. The nuclei were stained with DAPI for 5 min. Subsequently, the stained cells were observed and photographed using Olympus BX53 fluorescence microscope.

### Transwell migration assay

We seeded 1×10^5^ cells in 150 μL DMEM medium without FBS into the upper chamber of the Transwell (Corning, NY, USA). We then added 700 mL DMEM medium with 10% FBS into the lower chamber. The Transwell chambers were incubated for 24 h in at 37° C. Then, the cells attached to the lower surface of the membrane were fixed with 70% methanol, stained with 1% crystal violet for 30 min, and imaged using Olympus BX53 fluorescence microscope.

### Tube formation assay

We coated 24-well plates with 50 μLMatrigel (BD Biosciences, Franklin Lake, NJ, USA) per well. The plates were then incubated at 37° C for 30 min. Then, we seeded HUVECs (1×10^5^ cells/well) into the Matrigel-coated wells for 24 h. The cultures were photographed using the Olympus BX53 fluorescence microscope and the number of branch points were counted using ImageJ software.

### Western blotting

Total cellular proteins were extracted using RIPA buffer. Protein concentrations were determined using the BCA protein assay kit (Beyotime Institute of Biotechnology). Equal amounts of proteins were separated using 10% sodium dodecyl sulfate-polyacrylamide gel electrophoresis, and the separated proteins were transferred onto polyvinylidene difluoride (PVDF) membranes (Millipore, Billerica, MA, USA). Then, the membranes were blocked in 5% skimmed milk in TBST at room temperature for 1 h. The blots were incubated at 4° C overnight with the following primary antibodies (Abcam, Cambridge, MA, USA): anti-VEGF (1:1000), anti-angiogenin-1 (1:1000), anti-MMP9 (1:1000), anti-MMP14 (1:1000), anti-p-Akt (1:1000), anti-Akt (1:1000), anti-p-ERK (1:1000), anti-ERK (1:1000), anti-VEGFC (1:1000), anti-β-actin (1:1000). Then, the blots were incubated with HRP-conjugated secondary antibodies (1: 5000) for 50 min at room temperature. Subsequently, blots were developed using ECL (Fdbio Science, Hangzhou, China).

### Isolation of GSCs and transfections

CD133 is a cell surface marker of human brain tumor stem cells and is commonly used to isolate the GSCs [51]. We sorted SHG44 cells by flow cytometry using fluorescence-tagged antibodies against CD133 to isolate CD133^+^ GSCs using protocols described previously by Wang et al. [20]. These GSCs were cultured in DMEM/F12 medium (Thermo Fisher Scientific) without penicillin-streptomycin, and transfected with miR-944 agomir or miR-NC using Lipofectamine™2000. The transfected GSCs were cultured in a humidified incubator at 5% CO_2_ and 37° C for 6 h. Subsequently, the medium was replaced by fresh stem cell medium.

### Isolation and characterization of GSC-derived exosomes

Exosomes were isolated from transfected GSCs by ultracentrifugation. Briefly, transfected GSCs were cultured for 48 h in DMEM/F12 medium supplemented with 10% exosome-depleted FBS (prepared by overnight ultracentrifugation at 120,000×g and 4° C). Then, conditioned media (CM) was collected and exosomes were isolated using Hieff™ Quick exosome isolation kit. The exosomes were resuspended in PBS and stored at -80° C. The particle size distribution of exosomes was analyzed by NTA using a nanoparticle tracking analyzer. Western blot analysis was performed to determine the expression levels of exosomal surface markers, CD63 and TSG101.

### Co-culture of exosomes and HUVECs

The exosomes (20 μg) were fluorescently labeled with PKH67 membrane dye (Sigma). Then, HUVECs were incubated with PKH67-labeled exosomes at 37° C for 24 h. HUVEC cells were stained with phalloidin-FITC to label F-actin. DAPI was used to stain the nuclei. Subsequently, stained HUVEC cells were observed, photographed under a confocal microscope (Carl Zeiss, Jena, Germany) and analyzed to determine internalization of exosomes.

### Dual luciferase reporter assay

We cloned wild-type (WT) and mutated (MT) 3’-UTRs of VEGFC into the pmirGLO luciferase plasmid (GenePharma) and co-transfected them into HUVECs with agomiR-944 or agomiR-NC using Lipofectamine2000. After 48 h of incubation, we analyzed firefly and Renilla luciferase (endogenous control) activities in the cell lysates using the dual-luciferase reporter assay system (Promega).

### Animal study

We purchased BALB/c nude mice (4-6 weeks old) from the Shanghai Laboratory Animal Center (Shanghai, China). The mice were randomly divided into two main groups: (1) mice were subcutaneously injected with 5 × 10^6^ SHG44 cells into the left flank of nude mice; the mice in this group were then divided into 2 further groups: control and agomiR-944 groups; (2) mice were subcutaneously injected with 5 x 10^6^ GSCs into the left flank of nude mice; the mice in this group were then divided into control, agomiR-944, and agomiR-NC groups. When the tumors reached about 200 mm^3^, we injected 50 nM agomiR-944 or 50 nM agomiR-NC directly into the tumors twice a week. Tumor volume was calculated every week using the following formula: V = (length x width^2^)/2. The mice were sacrificed 3 weeks after the agomiR-944 or agomiR-NC injections. The tumors were harvested and used for further experiments. The animals were maintained according to the guidelines of the Institutional Animal Care and Use Committee. All animal experiments were approved by the Institutional Ethical Committee of the Taizhou People’s Hospital.

### Microvessel density (MVD) analysis

The tumors were fixed in 4% paraformaldehyde, embedded in paraffin, and then cut into 3 μm thick sections. The sections were incubated with DBE-conjugated anti-CD31 antibody (Abcam) at 4 ° C overnight, followed by incubation with the corresponding secondary antibody at room temperature for 1 h. Subsequently, stained tumor sections were imaged using Aperio Scan-Scope AT Turbo (Aperio, Sausalito, CA, USA).

### Statistical analysis

Statistical data was analyzed using the GraphPad Prism software version 7.0 (GraphPad Software Inc. San Diego, CA, USA). Differences between two groups were analyzed by Student’s t-test, whereas, differences between multiple groups were analyzed using one-way analysis of variance (ANOVA) followed by Tukey’s test. All experiments were performed at least thrice. All data are shown as means ± SD. P < 0.05 was considered statistically significant.

## Supplementary Material

Supplementary Figure 1
