# Supplementary material for: Glioma stem cell-derived exosomal miR-944 reduces glioma growth and angiogenesis by inhibiting AKT/ERK signaling
Source: Aging (Albany NY). 2021 Jul 7;13(15):19243–59. doi: 10.18632/aging.203243 (PMC8386563; doi:10.18632/aging.203243)
Supplement: Supplementary Figure 1 [file aging-13-203243-s001.pdf]

SUPPLEMENTARY FIGURE

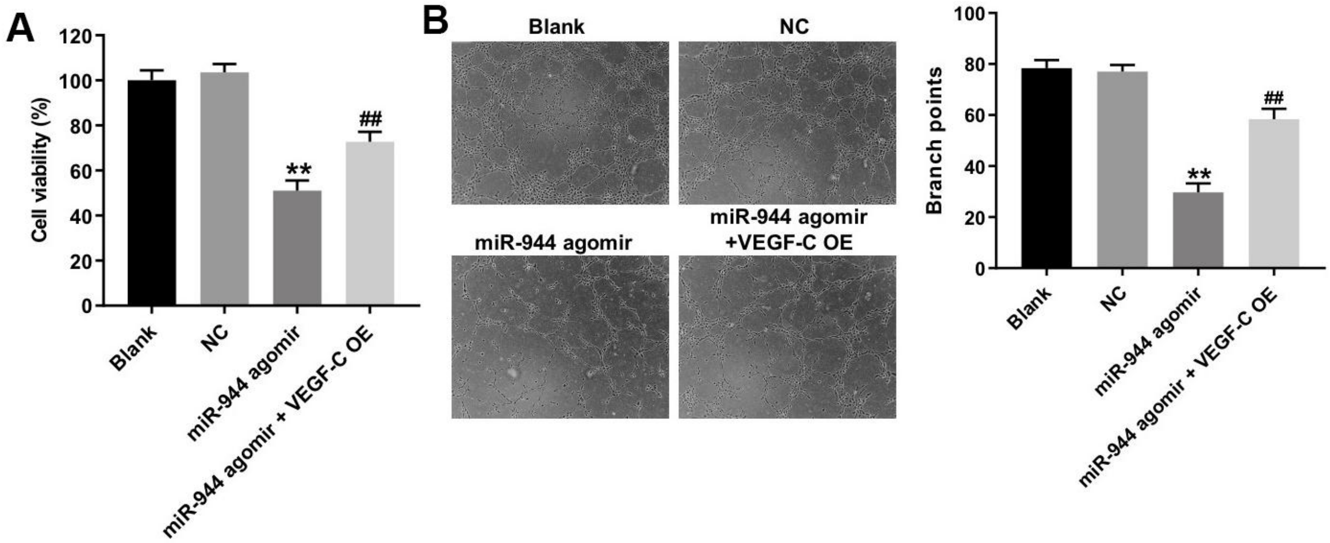

Supplementary Figure 1. Overexpression of miR-944 inhibits the viability and angiogenesis of HUVECs via downregulation of VEGFC. (**A**) CCK-8 assay results show viability of blank control, NC, miR-944 agomir-, and miR-944 agomir + VEGF-C-OE-transfected HUVECs. (**B**) Tube formation assay results show the number of branch points as an index of angiogenesis in blank control, NC, miR-944 agomir-, and miR-944 agomir + VEGF-C-OE-transfected HUVECs. **P < 0.01 vs. NC group; **P < 0.01 vs. miR-944 agomir group.
